# Vasculogenic mimicry in non-small cell lung cancer: a systematic review

**DOI:** 10.3389/fonc.2025.1481726

**Published:** 2025-07-25

**Authors:** Valeriia Shapkina, Vadim Shindyapin, Nikita Burlov, Elizaveta Prosekina, Anna Artemyeva

**Affiliations:** ^1^ Laboratory of Microangiopathic Mechanisms of Atherogenesis, Institute of Medicine, Saint-Petersburg State University, Saint Petersburg, Russia; ^2^ Department of Pathology, Institute of Medicine, Saint-Petersburg State University, Saint Petersburg, Russia; ^3^ Division of Immunobiology and Biomedicine, Scientific Center for Genetics and Life Sciences, Sirius University of Science and Technology, Krasnodarskiy kray, Russia; ^4^ Engelhardt Institute of Molecular Biology, Russian Academy of Sciences, Moscow, Russia; ^5^ Department of surgery, City Hospital, vil. Svobodniy, Sverdlovsk region, Russia; ^6^ Pathology department, Federal State Budgetary Institution "National Medical Research Center of Oncology named after N.N. Petrov" of the Ministry of Health of the Russian Federation, Saint Petersburg, Russia; ^7^ Laboratory of Tumor Morphology, Federal State Budgetary Institution "National Medical Research Center of Oncology named after N.N. Petrov" of the Ministry of Health of the Russian Federation, Saint Petersburg, Russia

**Keywords:** vasculogenic mimicry, non-small cell lung cancer, prognosis, histology, systematic review

## Abstract

Vasculogenic mimicry (VM), a non-endothelial tumor blood supply mechanism linked to poor prognosis in various cancers, requires consolidated prognostic evaluation in non-small cell lung cancer (NSCLC). This systematic review synthesized evidence on VM’s association with survival outcomes (OS, DFS, PFS) in NSCLC patients. Following PRISMA-ScR guidelines, PubMed and Google Scholar were searched, identifying 19 eligible studies (all in Chinese populations) using immunohistochemistry (CD31/CD34-PAS) for VM detection. Eighteen studies found VM presence (prevalence 13.6%–45.2%) significantly associated with worse survival. Multivariate analyses identified VM as an independent negative prognostic factor, increasing mortality risk (HR 1.542–2.542) and progression risk (HR 2.1–2.4). However, critical limitations included exclusive focus on Asian cohorts, universal retrospective design, inconsistencies and potential artifacts in VM detection, and statistical issues (misreported risk measures, discordant data). While VM correlates with reduced survival in NSCLC, suggesting potential prognostic utility, these limitations - particularly ethnic homogeneity, retrospective bias, methodological heterogeneity, and statistical errors - preclude definitive conclusions. Future prospective studies with standardized VM assessment and diverse populations are essential for validation.

## 1 Introduction

The phenomenon of VM represents a fundamentally new pattern of tumor blood supply in which tumor cells adapt to the microenvironment by acquiring the ability to form vascular structures functionally similar to blood capillaries (1). This ability of tumor cells to mimic endothelial cells and form vessel-like structures without the participation of endothelial cells demonstrates their plasticity and represents an evolutionary adaptation to hypoxic conditions and the need for blood supply within the tumor.

VM represents a key mechanism driving progression and therapy resistance in NSCLC. Despite advances in targeted and immune therapies, the five-year survival rate for NSCLC remains low, partly due to the formation of alternative tumor blood supply systems, including VM. Recent studies demonstrate that VM is associated with activation of HIF-1α, DDAH1, EMT and Wnt/β-catenin signaling pathways, which enhance tumor cell invasiveness and promote metastasis (2–4).

The clinical relevance of VM is underscored by the development of “VM-scores” based on *EPHA2*, *LAMC2*, and *LOXL2* genes, which serve as independent prognostic markers in lung adenocarcinoma (2). High VM scores correlate with an immunosuppressive tumor microenvironment, increased infiltration of neutrophils and specific immune cells (activated memory CD4 T cells, resting NK cells, M0 macrophages), while showing negative correlation with naïve B-cells. These tumors demonstrate elevated expression of immune checkpoints and higher TMB. Importantly, high VM scores are associated with increased sensitivity to several therapeutic agents including docetaxel, cisplatin, gemcitabine, paclitaxel, vinblastine, sorafenib and pazopanib. Targeting VM through doxazosin or PD-L1 suppression has shown promise in preclinical NSCLC models, offering new avenues for combination therapies (5, 6).

Critical molecular markers associated with VM include vascular endothelial-cadherin, EphA2, and matrix metalloproteinases such as MMP-2 and MMP-9, which facilitate cell-cell adhesion and extracellular matrix remodeling necessary for tubular network formation (1, 7, 8). In NSCLC, molecular features include the association of cancer stem cell markers like CD44 and nestin with VM, indicating a potential role in tumor vascularization and cellular plasticity (9). In contrast to melanomas, where VM is driven predominantly by Notch signaling (10), NSCLC VM demonstrates stronger dependence on EGFR-mediated pathways and HIF-1α stabilization (11). This distinction may be attributed to the high prevalence of EGFR mutations in lung adenocarcinoma, which activate downstream PI3K/AKT and MAPK pathways promoting VM formation.

Recent evidence highlights critical roles of growth factor networks in VM regulation. PD-L1, beyond its immune checkpoint function, directly promotes VM in NSCLC through ZEB1-mediated EMT activation, with knockdown reducing tube formation by 62% and suppressing VE-cadherin/MMP9 expression (6). Thrombin induces VM via PAR-1 receptor signaling, creating coagulation factor-driven vascular networks that correlate with reduced overall survival (HR=2.1, p<0.01) (12). The VEGF-A/VEGFR-2 axis remains important, with data showing doxazosin inhibits VM by 73% through VEGF-A suppression and mTOR/MMP-9 pathway blockade (5). Emerging evidence implicates TFPI-2 as a matrix-derived regulator of VM through MMP-2 modulation (13).

Molecular assessment now extends beyond immunohistochemistry to include methods like 3D Matrigel quantification of tube formation capacity (14) - a well-established and routine assay that evaluates the ability of cell cultures to form VM-like structures *in vitro*. In recent years, a number of publications have emerged utilizing omics technologies that introduce surrogate markers for VM detection and describe specific potentially targetable elements of its development:

1. VM-Score gene signatures (*EPHA2/LAMC2/LOXL2*) validated in TCGA lung adenocarcinoma cohorts (6, 15)
2 siRNA screening platforms identifying PD-L1/ZEB1 as druggable VM targets (6)
3. Multi-omics profiling revealing VM-associated immune checkpoint upregulation (PD-L1/CTLA-4/TIM-3) (15)

VM morphologically can be divided into two main types: tubular type and patterned matrix type. The tubular type is characterized by the formation of structures morphologically resembling ordinary blood vessels with a lumen, while the patterned matrix type is characterized by the formation of more complex and distinct patterns. Such structures include an organized network of channels and lagoons formed by cancer cells that integrate into the three-dimensional extracellular matrix networks. Blood and nutrients can be transported through these vessel-like structures, providing optimal conditions for the growth and dissemination, even though they may lack well-defined lumens.

This complex vascular phenomenon was first discovered by Maniotis and colleagues (16) in 1999 while studying uveal melanoma. Since then, this phenomenon has been detected in multiple malignancies, including breast cancer, melanoma, hepatocellular carcinoma, and, notably, NSCLC. A number of studies have demonstrated a correlation between the presence of VM phenomena and an increased level of tumor aggressiveness, their ability to grow and metastasize, as well as poor patient outcomes.

Building upon these discoveries, researchers have made significant progress in understanding the mechanisms and role of VM in the development and progression of tumor diseases, there are still a number of unresolved issues related to both the molecular biological aspects of this phenomenon and the possibility of using knowledge about VM to develop new therapeutic approaches. Understanding the specifics of VM in the context of NSCLC and its relationship to patient survival may help in finding new targets for drug therapy (17) and developing more effective antiangiogenic strategies for the treatment of these tumors (18).

Particularly in the context of NSCLC, a common and often aggressive form of cancer, the analysis of the VM phenomenon and its prognostic importance can greatly influence treatment and management approaches. Targeted and immune therapies targeting the unique pathways and mechanisms underlying VM can offer new perspectives for improving treatment effectiveness and outcomes for patients with NSCLC.

VM has been found in a variety of tumors of various localizations – mostly malignant (19–22), and sometimes benign (23).

Currently, 2 meta-analyses have been published on VM in tumors of various localizations (24, 25). Meta-analyses have shown that VM worsens the prognosis. However, no systematic reviews focusing on the role of VM in NSCLC have been published.

## 2 Materials and methods

### 2.1 Inclusion criteria

Initially, a comprehensive literature search was conducted to identify publications pertaining to VM for inclusion in the study. The primary inclusion criterion was that a substantial portion of the publication must be dedicated to investigating the phenomenon of VM in the context of NSCLC. Furthermore, the selected studies were required to evaluate the correlation between the presence of VM and patient survival outcomes, including overall survival (OS), disease-free survival (DFS), or progression-free survival (PFS). The literature search encompassed publications in both English and Chinese languages. For articles published in Chinese, an online translation tool was employed to facilitate the comprehensive analysis of the content.

### 2.2 Search

The study used the PRISMA-ScR algorithm to ensure a systematic approach. The literature search was performed using the PubMed and Google Scholar databases, with the specific queries detailed in the text below.

Search requests: **V.Shi., PubMed:**

(“2000”[Date - Publication]: “3000”[Date - Publication]) AND (((Carcinoma, Non-Small-Cell Lung[Mesh]) OR (Adenocarcinoma of Lung[Mesh]) OR ((Carcinoma, Large Cell[Mesh]) AND (lung[Title/Abstract])) OR ((Carcinoma, Squamous Cell[Mesh]) AND (lung[Title/Abstract])) OR (NSCLC[Title/Abstract]) OR (Nonsmall Cell Lung Cancer[Title/Abstract]) OR (Non-Small-Cell Lung Carcinoma[Title/Abstract]) OR (Non-Small Cell Lung Carcinoma[Title/Abstract]) OR (Non-Small Cell Lung Cancer[Title/Abstract]) OR ((Adenocarcinoma[Title/Abstract]) AND (lung[Title/Abstract])) OR ((Large cell carcinoma[Title/Abstract]) AND (lung[Title/Abstract])) OR ((Squamous cell carcinoma[Title/Abstract]) AND (lung[Title/Abstract]))) AND ((vascular mimicr*[Title/Abstract]) OR (vasculogenic mimicr*[Title/Abstract])))

**V.Sha., PubMed:**

((vasculogenic mimicry) or (vascular mimicry)) and ((nsclc) or (lung))

**V.Shi., Google Scholar:**

((«vascular mimicry» OR «vasculogenic mimicry») AND (“NSCLC” OR “non-small cell lung carcinoma” OR “non-small-cell lung carcinoma” OR “non-small cell lung cancer” OR “non-small-cell lung cancer” OR ((«Adenocarcinoma» OR «Large cell carcinoma» OR «Squamous cell carcinoma») AND «Lung»))) + date filter from 2000 till 2024

**V.Sha., Google Scholar:**

vasculogenic mimicry NSCLC

**Figure 1:**
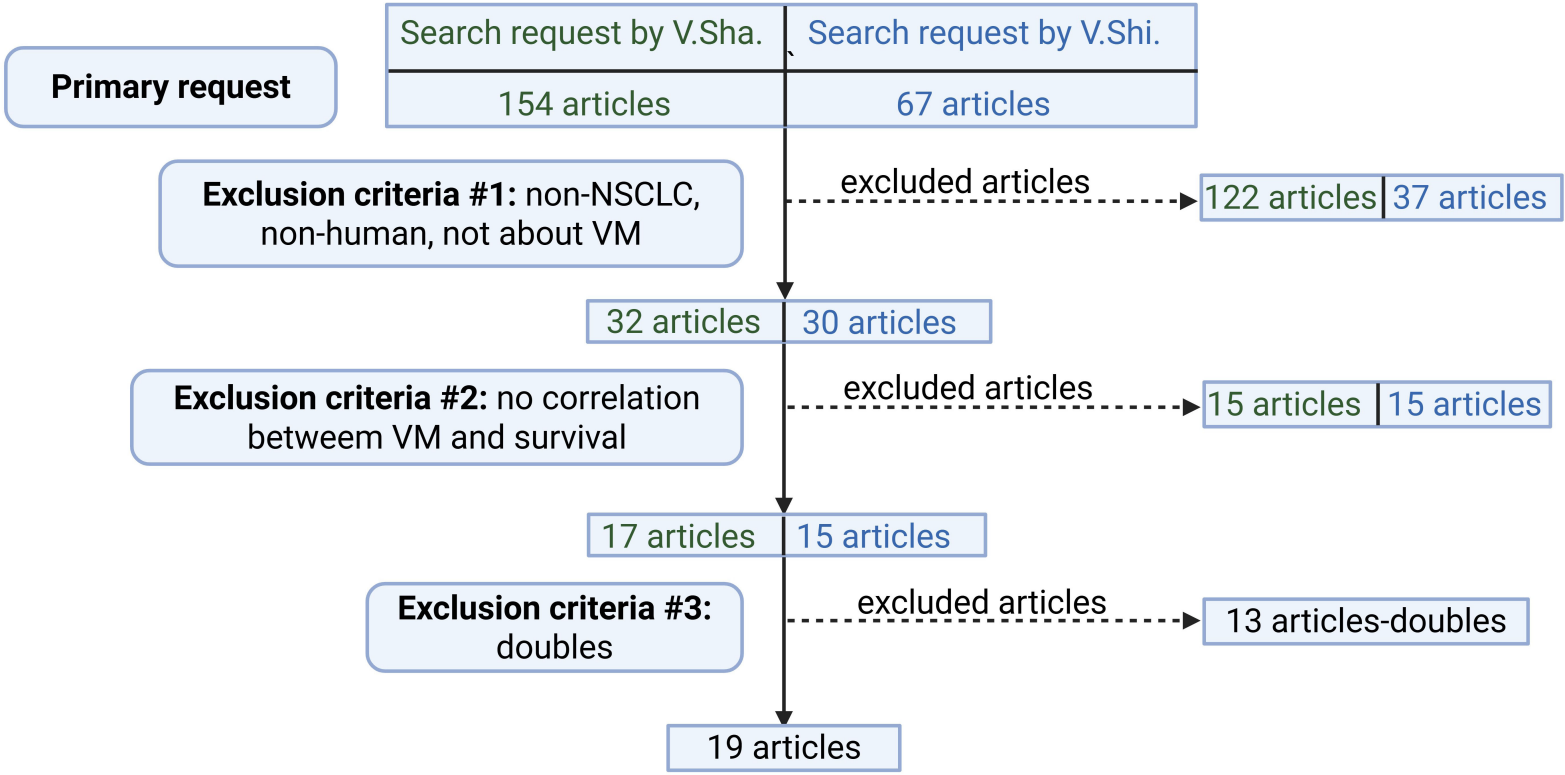
Study selection process. Created with http://www.BioRender.com.

The screening process of the articles was conducted independently by two researchers (V.Shi. and V.Sha.) to maintain objectivity and minimize bias. Both authors also performed cross-verification of search strategies, independently reviewing search terms and their application across databases to ensure comprehensive coverage of the literature. In controversial cases, the articles were subjected to a collective discussion with the involvement of a third researcher (N.B.) to reach a consensus (**Figure 1**).

### 2.3 Data extraction

The following data was collected:

- VM detection method;
- the source of tissues for research (biopsy or surgical resection);
- the use of tissue matrices;
- the number of estimated fields of view;
- staining methods for detecting VM (antigens for IHC - CD31 or CD34, PAS staining, combined staining).

To assess the methodological features of the studies under consideration, the following criteria were considered:

- design of the conducted research (prospective/retrospective);
- did the researchers align the groups according to TNM, demographic indicators, histological type and other characteristics;
- what kind of data on patient survival were evaluated.

### 2.4 Histological analysis and image acquisition

**Figure 2:**
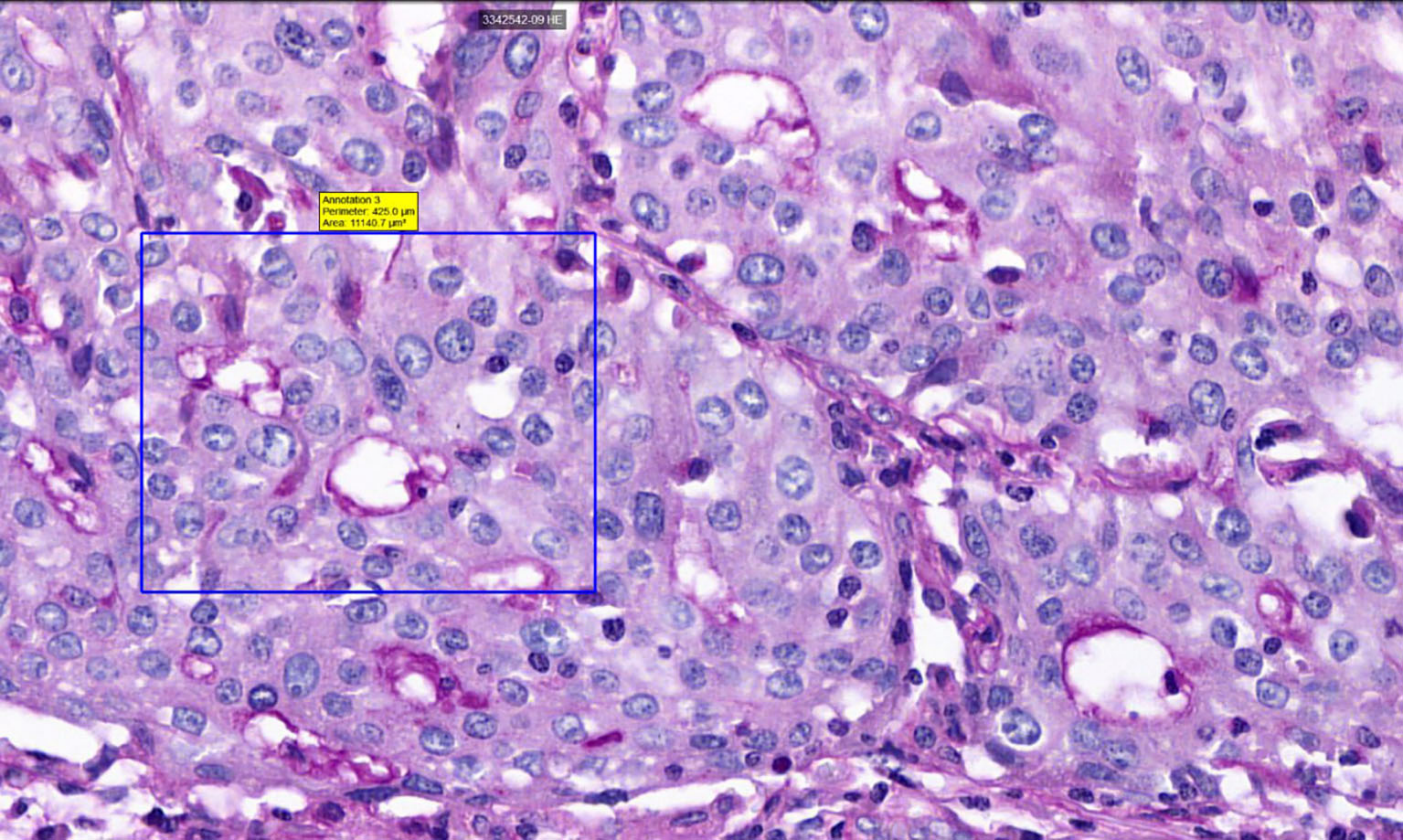
Histological sample of NSCLC with VM: tubular type, PAS staining, 40x magnification. A square indicates PAS-positive tubular VM structures, demonstrating characteristic PAS reactivity in vasculogenic mimicry patterns.

**Figure 3:**
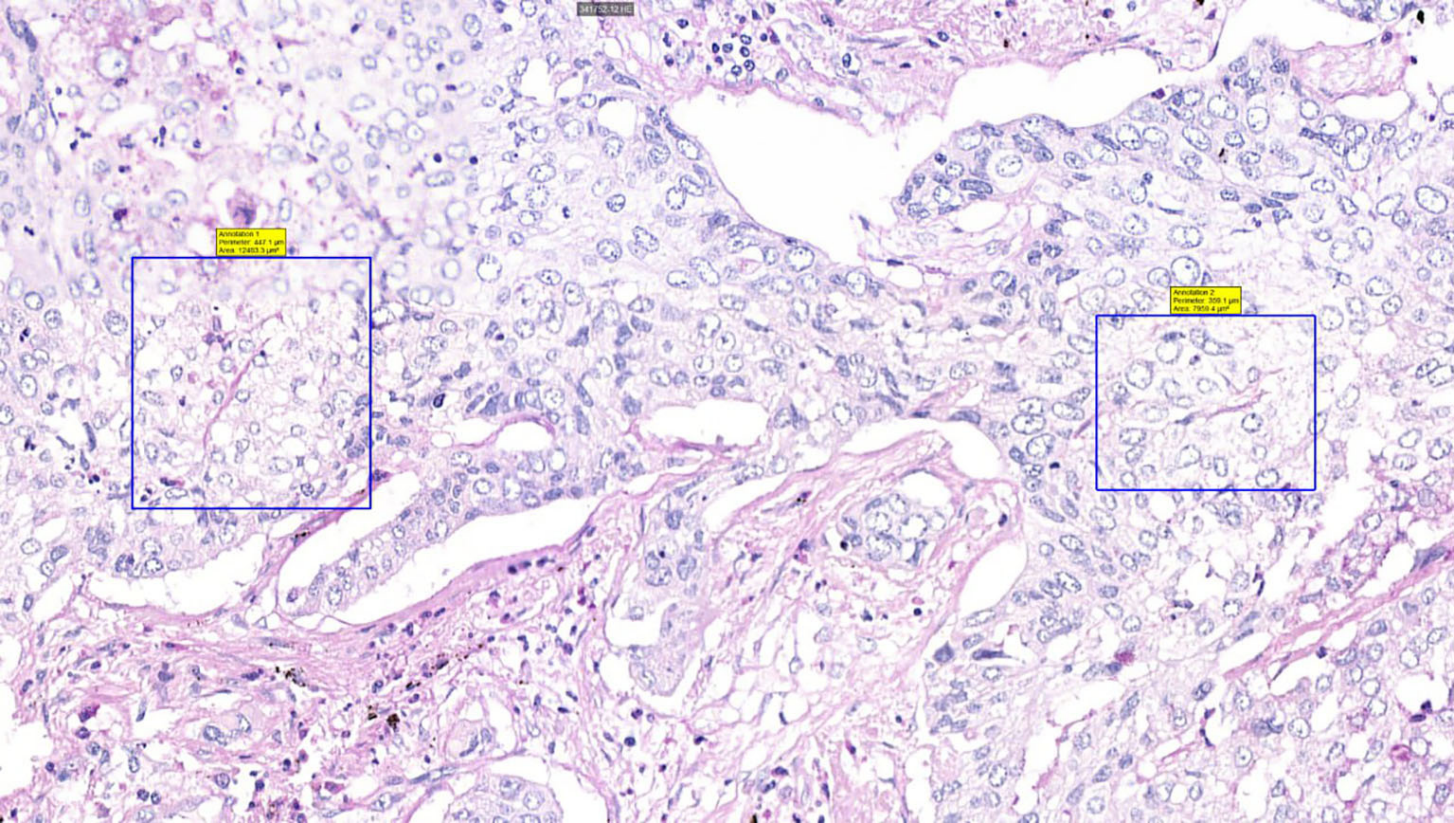
Histological sample of NSCLC with VM: patterned matrix type, PAS staining, 40x magnification. Squares highlight PAS-positive extracellular matrix sheets consistent with patterned matrix-type VM, demonstrating distinctive PAS reactivity in matrix-rich VM architectures.

**Figure 4:**
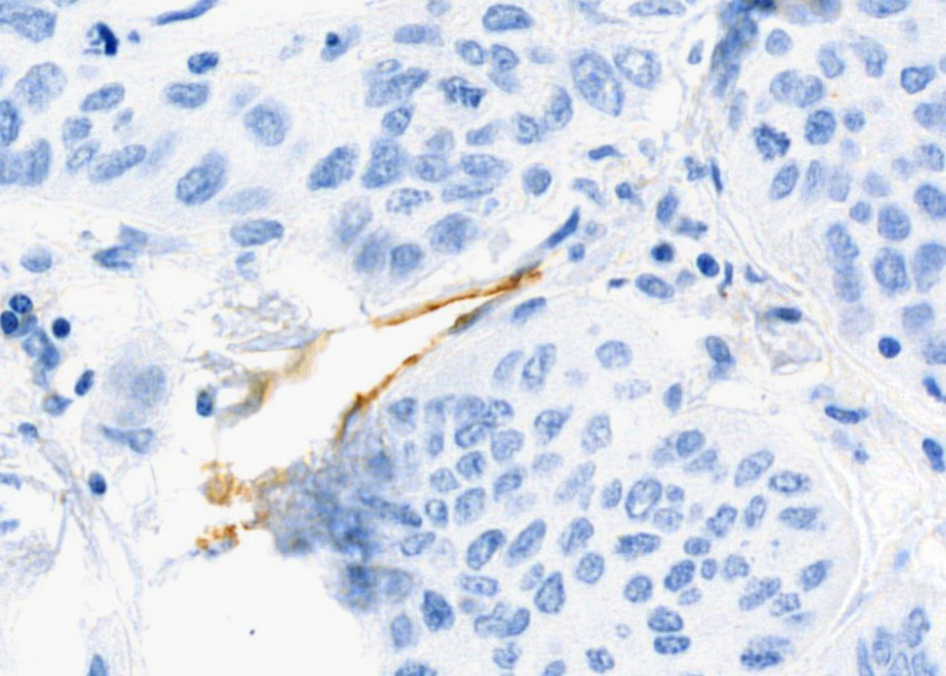
Histological sample of NSCLC with blood vessels: IHC staining for CD31, 400x magnification. Brown chromogenic staining highlights the vascular endothelium.

Surgically resected samples of NSCLC, formalin-fixed and paraffin-embedded, were stained with PAS to visualize VM. **Figures 2**, **3** were captured using the Pannoramic Confocal microscope (3D Histech) at 40x magnification. **Figure 4** depicts blood vessels in NSCLC tissue, identified via IHC for CD31 at 400x magnification. Brown chromogenic staining highlights the vascular endothelium.

**Figure 5:**
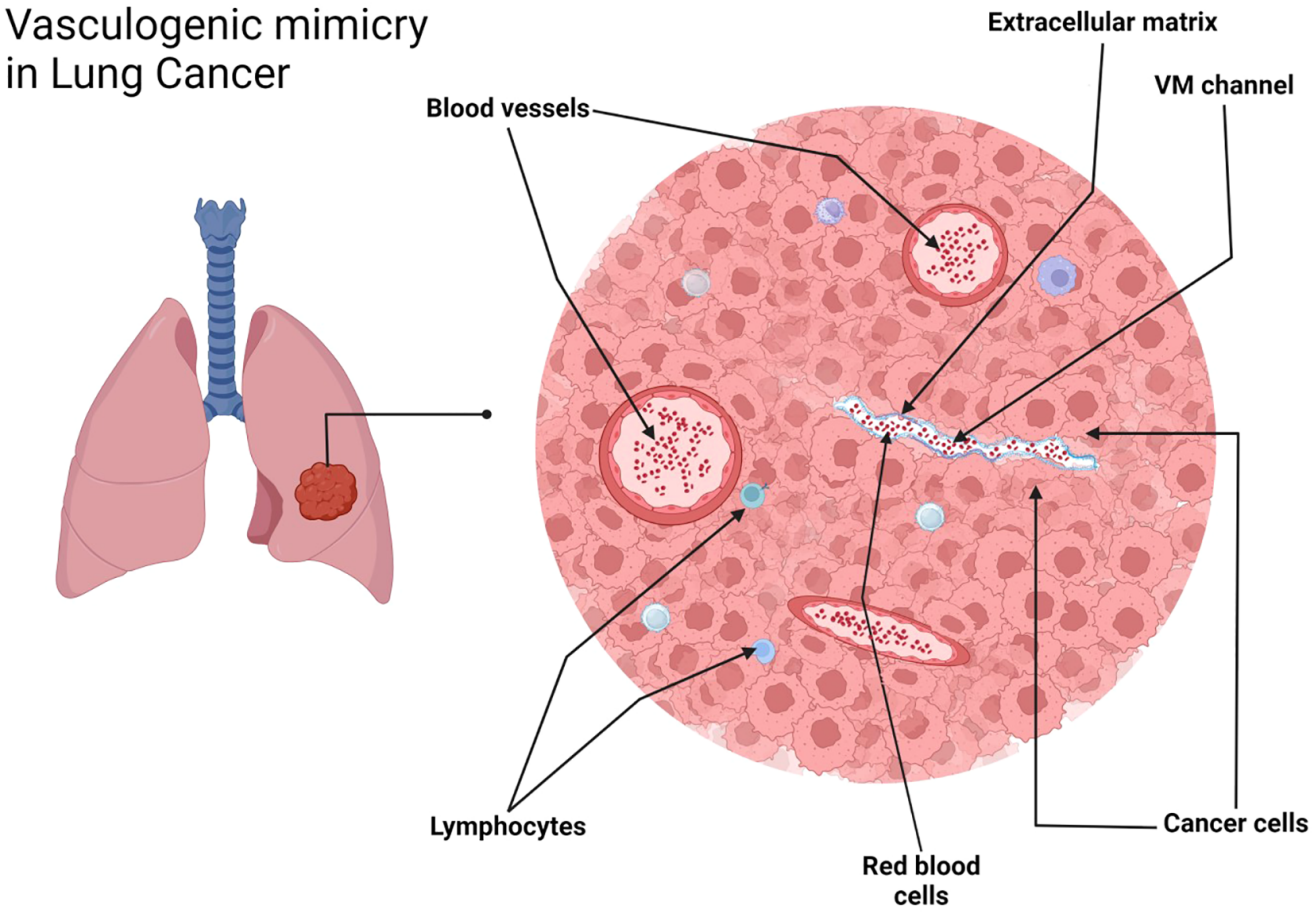
VM in NSCLC, tubular type. Schematic representation. Created with http://www.BioRender.com.

**Figure 6:**
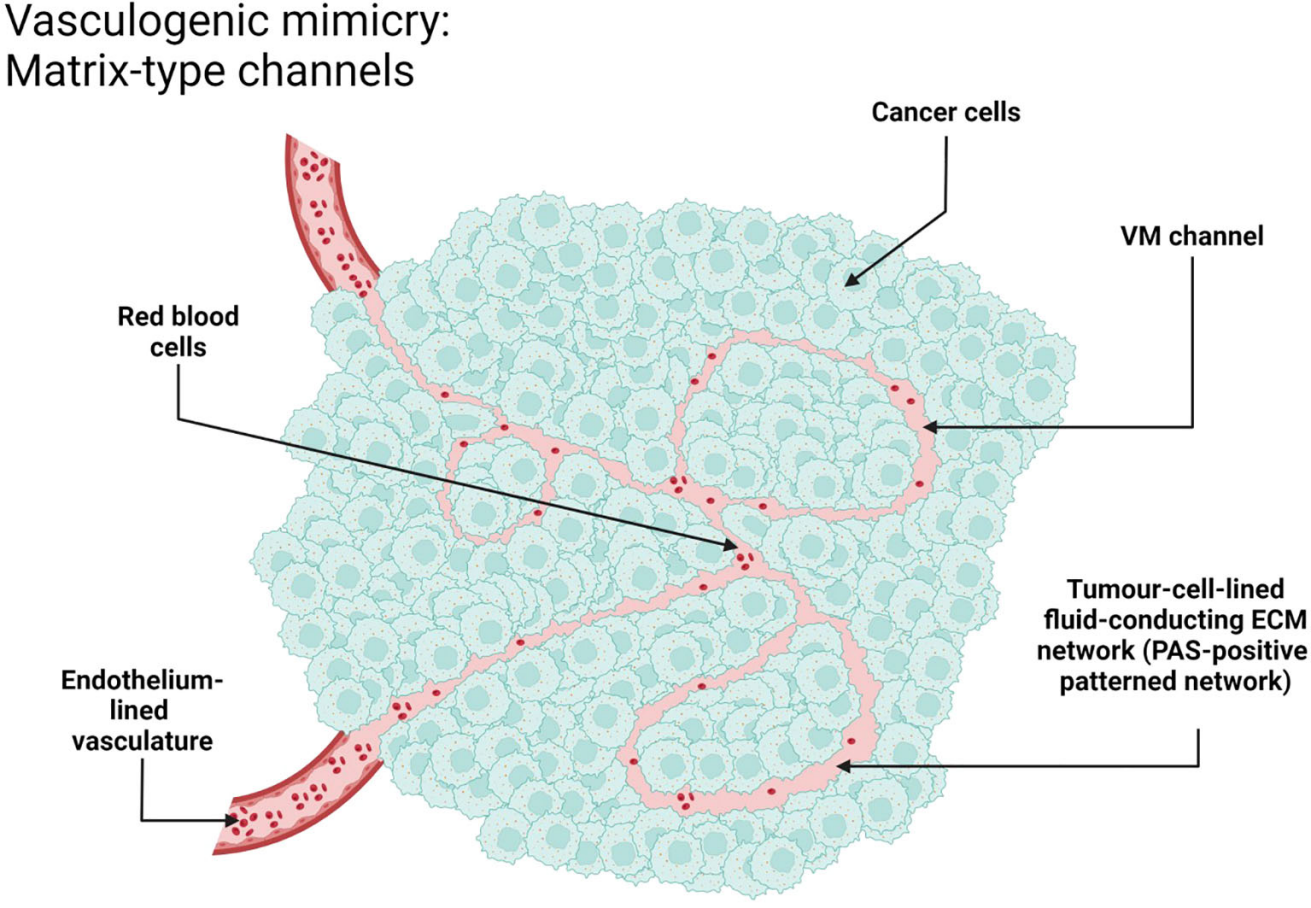
VM in NSCLC, patterned matrix type. Schematic representation. Created with http://www.BioRender.com.

**Figures 1**, **5**, **6** were schematic illustrations created with BioRender.com, depicting key aspects of the tubular and patterned matrix types of VM. Images and schematics were analyzed to assess mimicry characteristics.

## 3 Results

The PubMed search yielded 154 results from V.Sha. and 67 results from V.Shi. After the selection process, 62 articles were retained. Subsequently, 30 articles were excluded due to non-compliance with the inclusion criteria, and 13 articles were identified as duplicates. Consequently, a total of 19 articles remained for further analysis (**Table 1**). The PubMed search was initially conducted in July 2024, an updated search was subsequently performed in June 2025 to ensure the inclusion of the most recent publications and maintain the currency of the literature review.

**Table 1:** List of publications included in the study, with information on the date of publication, number of patients, place of study, study design, confounding factors, VM detection methods and endpoints.

Title	DoP	NoP	Place of research	Study design	Confounding factors	VM detection method	Endpoints
The role of TOP2A in immunotherapy and vasculogenic mimicry in non-small cell lung cancer and its potential mechanism	2023	141	First Affiliated Hospital of Bengbu Medical College	cohort	confounding factors not mentioned	CD31+ PAS, double	overall survival
Relationship between the expression of ARHGAP25 and RhoA in non-small cell lung cancer and vasculogenic mimicry	2022	130	First Affiliated Hospital of Bengbu Medical College	cohort	confounding factors not mentioned	CD34+ PAS, double	overall survival, disease-free survival
The Relationship Between UBE2C and AGGF1 Overexpression and Tumor Angiogenesis in Non-Small Cell Lung Cancer	2021	154	First Affiliated Hospital of Bengbu Medical College	cohort	confounding factors not mentioned	CD34+ PAS, double	overall survival, disease-free survival
The Angiotensin-Converting Enzyme Inhibitory State Promotes the Transformation of Non-Small Cell Lung Cancer Blood Supply Pattern Toward Vasculogenic Mimicry Formation	2021	83	Ruijin hospital	cohort	patients with similar age, gender, race, BMI, comorbidities etc.	CD34+ PAS, double, tissue TMAs were used	overall survival
Vasculogenic mimicry, a negative indicator for progression free survival of lung adenocarcinoma irrespective of first line treatment and epithelial growth factor receptor mutation status	2021	131	Second People’s Hospital of Taizhou City	cohort	patients with similar ECOG status and TNM stage.	CD34+ PAS. biopsies, 5 fields of view (magnification not specified)	progression-free survival
Thrombin is a therapeutic target for non-small-cell lung cancer to inhibit vasculogenic mimicry formation	2020	152	Fudan University Shanghai Cancer Center	Retrospective	confounding factors not mentioned	CD31+ PAS, double	overall survival
Evaluation of the correlation of vasculogenic mimicry, Notch4, DLL4, and KAI1/CD82 in the prediction of metastasis and prognosis in non-small cell lung cancer	2018	189	First Affiliated Hospital of Bengbu Medical College	cohort	confounding factors not mentioned	CD34+ PAS, double	overall survival
Expressions of KAI1 and E-cadherin in non-small cell lung cancer and their correlation with vasculogenic mimicry	2018	163	First Affiliated Hospital of Bengbu Medical College	cohort	confounding factors not mentioned	CD34+ PAS, double	overall survival
Vasculogenic mimicry and expression of Twist1 and KAI1 correlate with metastasis and prognosis in lung squamous cell carcinoma	2017	157	First Affiliated Hospital of Bengbu Medical College	cohort	confounding factors not mentioned	CD34+ PAS, double	overall survival
Dickkopf-1-promoted vasculogenic mimicry in non-small cell lung cancer is associated with EMT and development of a cancer stem-like cell phenotype	2016	205	Tianjin Medical University Cancer Institute and Hospital	cohort	confounding factors not mentioned	CD34+ PAS, double	overall survival
Overexpression of Wnt5a promotes angiogenesis in NSCLC	2015	205	Tianjin Medical University Cancer Institute and Hospital	cohort	confounding factors not mentioned	CD31+ PAS, double	overall survival, VM correlated with the expression of Wnt5a; Wnt5a correlated with overall survival
Aberrant expression of CD133 in non-small cell lung cancer and its relationship to vasculogenic mimicry	2012	305	First Hospital Affiliated to Bengbu Medical College	cohort	confounding factors not mentioned	CD34+ PAS, double	overall survival
Expression of maspin in non-small cell lung cancer and its relationship to vasculogenic mimicry	2012	160	First Hospital Affiliated to Bengbu Medical College	cohort	confounding factors not mentioned	CD34+ PAS, double	overall survival
Expression of CD82/KAI1 and HIF-1α in non-small cell lung cancer and their relationship to vasculogenic mimicry	2011	160	First Hospital Affiliated to Bengbu Medical College	cohort	confounding factors not mentioned	CD34+ PAS, double	postoperative survival time, 5-year survival rate
Vasculogenic mimicry and cancer stem-like cell markers are associated with poor prognosis of non-small cell lung cancer	2016	205	Tianjin Medical University Cancer Institute and Hospital	cohort	confounding factors not mentioned	CD31+ PAS, double	overall survival
The EMT transcription factor, Twist1, as a novel therapeutic target for pulmonary sarcomatoid carcinomas	2020	120	Tianjin Medical University	cohort	confounding factors not mentioned	CD31+ PAS, double	overall survival
Subpopulations of uPAR+ contribute to vasculogenic mimicry and metastasis in large cell lung cancer	2015	51	Tianjin Medical University Cancer Institute	cohort	confounding factors not mentioned	CD31+ PAS, double	mean survival
Vasculogenic mimicry and expression of slug and vimentin correlate with metastasis and prognosis in non-small cell lung cancer.	2018	198	First Affiliated Hospital of Bengbu Medical College	cohort	confounding factors not mentioned	CD34+ PAS, double	OST
Effects of Vasculogenic Mimicry on Postoperative Recurrence and Progression of Non-Small Cell Lung Cancer	2021	80	Taizhou Second People’s Hospital	single-center, cross-sectional study	confounding factors not mentioned	CD34+ PAS, double	recurrence-free survival

DoP, date of publication; NoP, number of patients.

The Google Scholar search conducted by V.Shi. researcher returned 3,880 articles, while the expanded search approach by V.Sha. yielded a substantially larger volume of initial results, indicating a need for refinement of search parameters. However, after systematic application of inclusion criteria, elimination of irrelevant sources, and removal of duplicates, no additional articles from these database searches met the criteria for inclusion in the final analysis.

Among the 19 selected articles, 9 originated from the First Affiliated Hospital of Bengbu Medical College (26–35). These articles utilized various sample sizes of NSCLC cases, ranging from 130 to 305 cases, spanning from 2003 to 2016. Notably, two of the nine articles (33, 34) employed identical sample populations. The samples encompassed NSCLC cases of the most prevalent histotypes, with adenocarcinomas and squamous cell carcinomas being the predominant subtypes.

The TNM stages reported in the articles ranged from stage I to stage IV. The authors did not provide descriptions of potential confounding factors. The studies included patients who didn’t undergo prior treatments such as chemotherapy, immunotherapy, radiation therapy, or the use of traditional Chinese medicine.

All studies were retrospective, cohort type. Potential confounding factors were not mentioned in any of the articles.

To detect VM, the authors used double staining – immunohistochemical (IHC) staining against CD34 or CD31 with subsequent PAS counterstaining. The study material consisted of surgical specimens, and the entire tissue blocks were subjected to thorough examination.

In the study “The role of TOP2A in immunotherapy and vascular mimicry in non-small cell lung cancer and its potential mechanism” there were 141 patients (26). The studies assessed OS as a primary outcome measure. Survival analysis, employing the log-rank test, revealed that VM-positive (VM+) patients experienced significantly poorer OS compared to VM-negative (VM-) patients (p<0.05). The median survival for the “VM+” group was approximately 26–28 months, whereas the “VM-” group demonstrated a notably longer median survival of around 54–56 months.

In the study «Relationship between the expression of ARHGAP25 and RhoA in non−small cell lung cancer and vasculogenic mimicry» there were 130 patients (27). The study evaluated both OS and DFS. The overall sample data revealed a median OS of 35 months and a 5-year survival rate of 20.9%. Patients with VM+ tumors exhibited significantly poorer OS compared to those with VM- tumors, with 5-year survival rates of 8% and 28.1%, respectively (log-rank test, p<0.001). Univariate analysis indicated that the presence of VM increased the risk of death by 2.7 times (univariate HR 2.655, 95% CI: 1.752-4.024, p<0.001). In a multivariate analysis, which included ARHGAP25, RhoA, lymph node metastasis (LNM), and TNM stage as covariates, the presence of VM increased the risk of death (multivariate HR 1.872, 95% CI: 1.195-2.933, p=0.006). Regarding DFS, VM+ patients demonstrated significantly worse outcomes compared to VM- patients, with 5-year DFS rates of 7.3% and 27.2%, respectively (log-rank test, p<0.001). Univariate analysis revealed that the presence of VM increased the risk of recurrence by 2.7 times (univariate HR 2.722, 95% CI: 1.814-4.084, p<0.001). In a multivariate analysis, adjusting for ARHGAP25, RhoA, LNM, and TNM stage, the presence of VM increased the risk of recurrence by 2 times (multivariate HR 1.958, 95% CI: 1.263-3.036, p=0.003).

In the study «The Relationship Between UBE2C and AGGF1 Overexpression and Tumor Angiogenesis in Non-Small Cell Lung Cancer» there were 154 patients (28). The study employed a cohort design, with relatively homogeneous groups. The primary outcomes assessed were OS and DFS. The overall sample data revealed a median OS of 41 months and a 5-year survival rate of 20.3%. VM+ patients exhibited significantly poorer OS compared to VM- patients (5-year survival rate: 2.2% vs. 30.2%, log-rank test p<0.001). Univariate analysis revealed that positive VM status increased the risk of mortality by 5.8-fold (univariate HR 5.773, 95% CI: 3.833-8.696, p<0.001). In the multivariate analysis, after adjusting for UBE2C, AGGF1, microvessel density (MVD), tumor size, LNM, and TNM stage, positive VM status remained an independent predictor of mortality, increasing the risk by 2.1-fold (multivariate HR 2.107, 95% CI: 1.226–3.622, p=0.007). Similarly, relapse-free survival was significantly worse in VM+ patients compared to VM- patients (5-year survival rate: 2.4% vs. 28.5%, log-rank test p<0.001). Univariate analysis demonstrated that positive VM status increased the risk of recurrence by 5.8-fold (univariate HR 5.802, 95% CI: 3.848-8.747, p<0.001). In the multivariate analysis, after controlling for the aforementioned covariates, positive VM status remained an independent predictor of recurrence, increasing the risk by 2.3-fold (multivariate HR 2.286, 95% CI: 1.333–3.920, p=0.003).

In a retrospective study titled “Evaluation of the correlation of vasculogenic mimicry, Notch4, DLL4, and KAI1/CD82 in the prediction of metastasis and prognosis of the course of non-small cell lung cancer” (29) a cohort of 189 patients was analyzed to assess the impact of VM on OS. The results demonstrated that the overall survival time (OST) was significantly shorter in patients with VM+ tumors (22.7 ± 13.3 months) compared to those with VM- tumors (52.3 ± 16.4 months; log-rank test = 126.642, P<0.001). The median survival time was approximately 21 months for VM+ patients and about 55 months for VM- patients.

In a study titled “Expressions of KAI1 and E-cadherin in non-small cell lung cancer and their correlation with vasculogenic mimicry” (30) 163 cases of NSCLC stages I to IIIa were investigated to determine the impact of VM on OS. The results revealed that the OS time of VM+ patients (21.86 ± 11.05 months) was significantly shorter than that of VM- patients (43.41 ± 17.10 months; log-rank test = 71.338, P<0.001). Patients with VM+ tumors had significantly worse OS compared to those with VM- tumors (p<0.001). The median survival time was approximately 20 months for VM+ patients and about 42–43 months for VM- patients. Furthermore, a multivariate analysis was performed, controlling for E-cadherin, KAI1, TNM stage, LNM, and tumor size. The results indicated that the presence of VM independently increased the risk of mortality by 62% (relative risk 1.621, 95% confidence interval [CI]: 1.032-2.547, p=0.036).

In a study titled “Vasculogenic mimicry and expression of Twist1 and KAI1 correlate with metastasis and prognosis in lung squamous cell carcinoma” (31) 157 cases of lung squamous cell carcinoma were investigated to determine the impact of VM on OS. The results demonstrated that the OST was significantly shorter in lung squamous cell carcinoma patients with VM+ tumors (32.6 ± 13.7 months) compared to those with VM- tumors (51.6 ± 12.1 months; log-rank test = 53.783, P<0.001). Patients with VM+ tumors had significantly worse OS compared to those with VM- tumors (p<0.001). The median survival time was approximately 32–33 months for VM+ patients and about 58 months for VM- patients. Moreover, a multivariate analysis revealed that the presence of VM was an independent prognostic factor for OS, with a hazard ratio (HR) of 2.348 (95% CI: 1.535-3.592, p<0.001), indicating that VM+ patients had a 2.348-fold increased risk of mortality compared to VM- patients.

In a study titled “Aberrant expression of CD133 in non-small cell lung cancer and its relationship to vasculogenic mimicry” (32) the authors investigated biopsies of 305 cases of NSCLC, including 210 squamous cell carcinoma and 95 adenocarcinoma, to determine the impact of VM on OS. The results demonstrated that patients with VM+ tumors had significantly worse OS compared to those with VM- tumors (p<0.001). The mean survival times were 19.0 months for the VM+ group and 58.1 months for the VM- group. However, it should be noted that there was a discrepancy in the presentation of the survival graphs, with the graphs for the dependence of survival on VM and CD133 expression being interchanged. Furthermore, a multivariate analysis was performed, controlling for CD133 expression, MVD, pathological TNM stage (pTNM), and therapy. The results indicated that the presence of VM independently increased the risk of mortality by approximately 1.9 to 2-fold (HR 1.914, 95% CI: 1.229-2.982, p=0.004).

In a study titled “Expression of maspin in non-small cell lung cancer and its relationship to vasculogenic mimicry” (33) 160 cases of NSCLC were investigated to determine the impact of VM on OS. The results demonstrated that patients with VM+ tumors had significantly poorer OS compared to those with VM- tumors (p<0.001). A multivariate analysis was performed, controlling for maspin expression, MVD, and pTNM. The results indicated that the presence of VM independently increased the risk of mortality by 2-fold (HR 2.046, 95% CI: 1.015–4.125, p=0.045). The mean survival time for VM+ patients was approximately 18 months, while for VM- patients, it was around 60 months. Furthermore, a significant correlation was observed between the presence or absence of VM and the prognostic implications in NSCLC patients (χ2 = 118.958, P=0.000), highlighting the potential of VM as a prognostic marker in NSCLC.

The article “Expression of CD82/KAI1 and HIF-1α in non-small cell lung cancer and their relationship to vascular mimicry” (34) was the one article from our sample, published in Chinese. The same sample of 160 NSCLC cases from the previous study (33) was investigated to determine the impact of VM on OS and its relationship with CD82/KAI1 and HIF-1α expression. A multivariate survival analysis was performed, and the results indicated that the presence of VM was an independent prognostic factor for OS in NSCLC patients. The HR for VM+ cases was 2.542 (95% CI: 1.422-4.543, p=0.002), suggesting that patients with VM+ tumors had a 2.542-fold increased risk of mortality compared to those with VM- tumors.

In a study titled “Vasculogenic mimicry and expression of slug and vimentin correlate with metastasis and prognosis in non-small cell lung cancer” (35) 198 cases of NSCLC were investigated to determine the impact of VM on OST and its relationship with the expression of slug and vimentin. The follow-up data revealed that the OST was significantly shorter in NSCLC patients with VM+ tumors (22.3 ± 13.3 months) compared to those with VM- tumors (41.9 ± 20.4 months; log-rank test = 56.100, P<0.001. Furthermore, a multivariate analysis was performed, controlling for slug expression, vimentin expression, LNM, distant metastasis, and TNM stage. The results demonstrated that the presence of VM independently increased the risk of mortality by 1.5-fold (HR 1.542, 95% CI: 1.025-2.320, p=0.038).

Four articles were affiliated with Tianjin Medical University Cancer Institute and Hospital (9, 36–38).

Three studies (9, 36, 37) were conducted on a sample of 205 patients diagnosed with NSCLC who underwent treatment between October 1990 and November 2010. The histological types of NSCLC in the sample were as follows: 79 cases of squamous cell carcinoma, 75 cases of adenocarcinoma, and 51 cases of large cell carcinoma. The study material was obtained from surgical specimens. The patients didn’t receive any prior treatment before undergoing surgical resection.

In another study (38) the researchers focused specifically on 51 cases of large cell carcinoma. We suppose the study sample to be derived from the same cohort of 205 NSCLC patients described in the previous three studies.

The studies included in all four articles were retrospective, and the authors did not describe potential confounding factors.

The detection of VM in these studies was performed using double CD31+PAS staining. However, the authors only considered cases displaying the tubular type of VM, which may not provide a comprehensive representation of all VM patterns. Furthermore, the VM images presented in three out of the four articles raise concerns about the accuracy of identification of VM. The structures that the authors considered to be VM could potentially be artifacts or glycogen inclusions within the cytoplasm of individual cell groups, rather than true VM formations.

The overall survival rate was assessed in three of the articles. Interestingly, two of these studies found no significant differences in survival between the VM+ and VM- groups. In contrast, the first article (36) reported a difference in the average survival period between the two groups. Patients with VM+ tumors had an average survival of 25.95 months, while those with VM- tumors had an average survival of 68.07 months (p=0.001). However, upon examining the Kaplan-Meier curves, it appears that the median survival for VM- patients is approximately 40 months, while for VM+ patients, it is around 10 months.

In the article titled “Vascular mimicry and cancer stem-like cell markers are associated with poor prognosis of non-small cell lung cancer” (9), the researchers reported a significant difference in the OS between patients with VM+ and VM- tumors. The average survival period for VM+patients was found to be 26 months, considerably shorter than the 68 months observed in VM- patients (p=0.001). It is important to note that the sample population in this article is likely the same as that in the previously mentioned study (36), as the patients data appear to be consistent between the two publications. The Kaplan-Meier survival analysis in this study demonstrated that patients with VM+ tumors had significantly worse OS compared to those with VM- tumors (p<0.001). The median survival time for VM+ patients was approximately 12–14 months, while for VM- patients, it was around 30–32 months.

In the article titled “Subpopulations of uPAR+ contribute to vascular mimicry and metastasis in large cell lung cancer” (38) the researchers attempted to estimate the mean survival time for patients with and without VM. They reported that the mean survival time for the VM+ group was 22.388 ± 5.220 months, which was significantly shorter than that of the VM- group (75.228 ± 12.218 months) (P = 0.020). However, it is important to note that the use of mean survival time may not be the most appropriate measure in this context due to the skewed nature of survival data. In survival analysis, the distribution of survival times is often right-skewed, with a few individuals having much longer survival times than the majority of the population. In such cases, the median survival time is generally considered a more robust measure, as it is less sensitive to extreme values and provides a better representation of the central tendency of the data. Upon examining the Kaplan-Meier curves presented in the article, it appears that the median survival time for VM+ patients is approximately 10 months, while for VM- patients, it is around 65 months.

2 investigations from the sample were performed in Second People Hospital of Taizhou City.

The first study (11), retrospective, was conducted on a sample consisting of 131 patients with advanced (stages IIIB and IV) EGFR-mutant adenocarcinoma who had not received prior treatment. The end point was PFS. The selected patients had similar TNM stages and ECOG status. The presence of VM was evaluated using CD34+PAS staining on biopsy material, with five fields of vision (the authors didn’t specify the magnification) examined for each sample. The study found that 45 patients were VM+, while 86 patients were VM-. Kaplan-Meier survival analysis revealed that VM+ patients had a significantly shorter median PFS of 167 days (range, 90-369; 95% CI: 138.14-195.86) compared to VM- patients, who had a median PFS of 279 days (range, 90-1095; 95% CI: 260.87-297.13). The difference in PFS between the two groups was statistically significant (log-rank test, p<0.001). Univariate Cox regression analysis showed that the presence of VM was associated with a 3.4-fold increased risk of disease progression (HR: 3.406; 95% CI: 2.299-5.047; p<0.001). In a multivariate analysis, adjusting for potential confounding factors such as sex, age, smoking status, T stage, N stage, M stage, differentiation, EGFR mutation, first-line therapy, and ECOG status, the presence of VM remained an independent predictor of shorter PFS, with a 2.1-fold increased risk of progression (HR: 2.118; 95% CI: 1.331-3.372; p=0.002).

The second study (39) was a single-center cross-sectional study. The sample included 80 untreated NSCLC patients (43 with adenocarcinoma and 37 with squamous cell carcinoma) diagnosed between March 2015 and February 2016, with stages ranging from I to IIIB. The primary endpoint was DFS, and the follow-up period was 3 years. VM was assessed in surgical specimens using IHC with CD34+PAS staining. VM was assessed in surgical specimens using IHC with CD34+PAS staining. The study found significant differences in mean recurrence-free survival (RFS) between VM+ and VM- groups. The mean RFS for the VM- group was 32 months, while for the VM+ group, it was 18 months. Multivariate analysis, adjusting for potential confounders such as gender, age, smoking status, histopathology, differentiation, T-stage, N-stage, and clinical stage, demonstrated that VM was an independent predictive factor for recurrence and progression. The presence of VM increased the risk of recurrence or progression by 2.4-fold (HR: 2.38; 95% CI: 1.16-7.2; p=0.002). The 3-year recurrence and progression rate were significantly higher in VM+ patients (83%) compared to VM- patients (20%) (p<0.001). The median 3-year recurrence-free survival was 32 months for VM- patients and 18 months for VM+ patients (p<0.001). Subgroup analysis revealed similar trends, with VM+ patients having shorter median RFS in both squamous cell carcinoma (VM-: 34.4 months; VM+: 18.9 months; p<0.001) and adenocarcinoma (VM-: 29.7 months; VM+: 17.5 months; p<0.001) subgroups.

In a study conducted by (40) at Ruijin Hospital, the prognostic significance of VM and angiotensin-converting enzyme 2 (ACE2) expression in NSCLC was investigated. The histological subtypes of the included NSCLC cases were not specified. The study included 83 NSCLC patients who underwent surgery between 2013 and 2016, with a follow-up period of 5 years. To minimize potential confounding factors, patients with similar characteristics such as age, gender, race, BMI, and comorbidities were recruited. Patients who survived less than one month or were lost to follow-up were excluded from the analysis. VM was assessed in surgical resection specimens using IHC with CD34+PAS staining on tissue microarrays constructed from tissue blocks. The study did not evaluate the impact of VM on survival independently; instead, patients were stratified into groups based on the expression levels of ACE2 in tumor cells (ACE2-low or ACE2-high). These groups were further divided into subgroups according to the presence or absence of VM in the tumors. In the ACE2-low expression group, patients with VM+ tumors had a significantly worse OS compared to those with VM- tumors (log-rank p=0.021). The median OS was approximately 19–21 months for the VM+ subgroup and 60 months for the VM- subgroup. Conversely, in the ACE2-high expression group, patients with VM+ tumors displayed a better OS compared to those with VM- tumors, although this difference was not statistically significant (log-rank p=0.179). The median OS was not reached in either subgroup.

One single-center retrospective study conducted by Fudan University (41) investigated a sample of 152 cases of NSCLC ranging from stage I to stage IV. The sample included 118 cases of adenocarcinoma, 25 cases of squamous cell carcinoma, and 9 cases of other types of NSCLC without specifying the histotypes. VM was assessed in surgical specimens using combined CD31+PAS staining. Interestingly, the authors described the criteria of detection of CD31-negative structures as VM and also scored for the number and size of areas with morphology consistent with mimicry. The criteria used were as follows: (1) the presence of PAS-positive channels that contained red cells and fluid, (2) the absence of CD31 staining in these channels, and (3) the polarization of tumor cells on an indistinct or imperceptible matrix lining vascular channels with red cells and/or fluid and no evidence of endothelization or tumor cells lining vascular spaces with no evidence of a matrix. The study estimated the TTR and 16-month survival. The authors calculated mean survival instead of median. Potential confounders weren’t described. Univariate survival analysis revealed that patients with VM had a significantly reduced 16-month survival (35.4 ± 4.8 months) compared to those without tumor VM (51.2 ± 5.2 months). Furthermore, patients exhibiting VM had a poorer prognosis and a higher likelihood of post-surgical relapse, with a time-to-recurrence of 20.0 ± 3.5 months, in contrast to 38.0 ± 5.3 months for patients without VM. Also, the study demonstrated that VM+ patients had significantly worse OS compared to VM- patients (p<0.0001). Kaplan-Meyer curves demonstrate median survival of approximately 35–36 months for VM+ patients and 61–62 months for VM- patients.

## 4 Discussion

VM was discovered in 1999 by Maniotis and colleagues. From the very beginning, the phenomenon itself raised a number of questions (42). Since that moment, clinical and morphological studies dedicated to the influence of VM on patient prognosis have been published.

Strangely enough, a large part of the research is devoted to melanoma. Not many articles are dedicated to NSCLC. At the time of writing this work, 19 articles have been published in the PubMed and Google Scholar databases, where the authors describe the correlation between the presence of VM and the prognosis of patients with NSCLC.

Currently, the prevalence of VM in tumors remains unclear, with different sources reporting varying figures. A positive correlation has been described between VM and increased T stage, presence of distant metastases and lymph node metastases, and higher tumor grade.

To date, two systematic reviews with meta-analyses have been published on VM, both describing the correlation between VM and survival across a range of tumor types, without focusing on a single nosology. No systematic reviews specifically investigating VM in NSCLC have been published thus far.

One of the challenges encountered is the issue of population. All studies included in this review were performed on material from Asian patients, with no published research apparently available on VM in NSCLC among European populations.

The detection of VM poses a challenge. Broadly, VM can be classified into two types: tubular type (**Figures 2**, **5**) and patterned matrix type (**Figures 3**, **6**). In contrast, **Figure 4** demonstrates CD31-positive endothelial-lined blood vessels in NSCLC, representing traditional angiogenesis-dependent vasculature.

The patterned matrix type has been most extensively described in the context of uveal melanoma. It is formed by clusters of melanoma cells wrapped in sheets of extracellular matrix, with spaces between them filled with blood plasma and sometimes blood cells.

Currently, there are no established guidelines, such as CAP protocols, for the detection of VM. The most commonly used method is double staining of tissue sections, combining IHC for endothelial markers such as CD31 or CD34 with PAS staining. VM structures are PAS-positive but do not express vascular markers.

Among the reviewed articles, 13 studies used CD34+PAS staining, and 6 - CD31+PAS staining.

Frequently encountered issues in VM detection include the differential diagnosis of VM from other similar phenomena, such as:

- Artifactual changes in the tumor arising during tissue processing and microtomy
- Poorly formed tumor glands
- Remnants of normal structures
- Small blood vessels
- So-called blood lakes
- Areas of necrosis
- Intracellular glycogen inclusions

The detection of patterned matrix-type VM presents a separate challenge. In this case, it is necessary to differentiate complex three-dimensional structures, located between cell clusters and composed of basement membrane proteins and glycoproteins, from collagen fibers. To address this, existing publications suggest staining the specimen with a trichrome stain (e.g., Masson’s trichrome) and/or performing morphometry of the structures. Basement membranes in VM are statistically significantly thinner than collagen fibers.

Surgical specimens are commonly used for the identification of VM. In our sample there were two exceptions: one study (40) used tissue microarrays, and another one (39) - biopsy samples.

In seven articles, the illustrations depicting VM raise doubts about the nature of the identified structures. In the article “Evaluation of the correlation of vasculogenic mimicry, Notch4, DLL4, and KAI1/CD82 in the prediction of metastasis and prognosis in non-small cell lung cancer” (29), the structure presented as VM could be considered a blood lake. In the articles “Dickkopf-1-promoted vasculogenic mimicry in non-small cell lung cancer is associated with EMT and development of a cancer stem-like cell phenotype” and “Overexpression of Wnt5a promotes angiogenesis in NSCLC,” intracellular glycoprotein inclusions are visible in the tissue sections (36, 37).

In the articles “Vasculogenic mimicry and expression of Twist1 and KAI1 correlate with metastasis and prognosis in lung squamous cell carcinoma”, “Aberrant expression of CD133 in non-small cell lung cancer and its relationship to vasculogenic mimicry”, “Vasculogenic mimicry and cancer stem-like cell markers are associated with poor prognosis of non-small cell lung cancer”, “Vasculogenic mimicry and expression of slug and vimentin correlate with metastasis and prognosis in non-small cell lung cancer” and “Dickkopf-1-promoted vasculogenic mimicry in non-small cell lung cancer is associated with EMT and development of a cancer stem-like cell phenotype” the authors might have mistaken artifactual changes or tumor growth patterns with cellular dropout for VM (9, 31, 32, 35, 36). Moreover, only one of the articles mentioned identification of patterned matrix-type. However, they didn’t attach illustrations of patterned matrix-type VM; only tubular VM was depicted.

The prevalence of VM in the samples varied dramatically, with minimal prevalence of 13,6% (9, 36, 37) and maximal - 45,2% (31).

In most of the articles included in this review, the presence of VM was demonstrated to be a negative prognostic factor. In studies where multivariate analysis was conducted, VM was shown to be an independent negative prognostic factor. The risk of mortality increased by 1.5 to 2.7 times, while the risk of disease progression increased by 2.1 to 2.4 times. The sole exception was the article titled “The Angiotensin-Converting Enzyme Inhibitory State Promotes the Transformation of Non-Small Cell Lung Cancer Blood Supply Pattern Toward Vasculogenic Mimicry Formation”. In this study, among patients with high tumor ACE expression, the VM+ subgroup showed better survival compared to the VM- subgroup; however, the result did not reach statistical significance (40).

Several articles presented methodological concerns related to the reporting and interpretation of statistical analyses. OS was not quantitatively reported in the following investigations: «The role of TOP2A in immunotherapy and vasculogenic mimicry in non-small cell lung cancer and its potential mechanism»; «The Angiotensin-Converting Enzyme Inhibitory State Promotes the Transformation of Non-Small Cell Lung Cancer Blood Supply Pattern Toward Vasculogenic Mimicry Formation» (26, 40).

In the articles «Evaluation of the correlation of vasculogenic mimicry, Notch4, DLL4, and KAI1/CD82 in the prediction of metastasis and prognosis in non-small cell lung cancer», «Expressions of KAI1 and E-cadherin in non-small cell lung cancer and their correlation with vasculogenic mimicry», «Vasculogenic mimicry and expression of slug and vimentin correlate with metastasis and prognosis in non-small cell lung cancer» the authors employed multivariate Cox proportional hazards regression analysis to assess the independent prognostic value of various factors (29, 30, 35). However, the tables in these studies report risk ratios instead of HR, which is inconsistent with the methodology typically used in survival analysis. HR, derived from the Cox regression model, are the appropriate measure for quantifying the effect of predictor variables on the hazard rate of an event occurring over time. The use of risk ratios in this context may lead to misinterpretation of the results and hinder the accurate assessment of the prognostic significance of the investigated factors.

In the article «Dickkopf-1-promoted vasculogenic mimicry in non-small cell lung cancer is associated with EMT and development of a cancer stem-like cell phenotype» the authors report statistically significant results in the text, stating that survival in the VM+ group is worse than in the VM- group (36). However, the presented Kaplan-Meyer curves appear to demonstrate the opposite trend. This discrepancy between the textual description and the graphical representation of the results raises concerns about the accuracy and reliability of the findings. Furthermore, the authors do not specify which type of survival they are referring to (e.g., OS, DFS, or PFS) when discussing the differences between the VM+ and VM- groups. Moreover, the authors use the term “average survival period”, which is not a standard measure in Kaplan-Meier survival analysis. In survival analysis, the appropriate measures to report include median survival time, survival rates at specific time points, and HR derived from Cox regression models.

In the article «Subpopulations of uPAR+ contribute to vasculogenic mimicry and metastasis in large cell lung cancer» the authors compare the mean OST (38). This approach is methodologically inappropriate for analyzing survival data.

In conclusion, a systematic search of the Pubmed and Google Scholar databases yielded 19 articles investigating the prognostic significance of VM in NSCLC. However, several methodological limitations and inconsistencies were identified across these studies, raising concerns about the reliability and generalizability of the findings.

Firstly, all the included studies were conducted on Asian populations, which may limit the applicability of the results to other ethnic groups. Secondly, the vast majority of the studies employed a retrospective design, which is prone to selection bias and confounding factors. Although most articles suggest that VM negatively impacts overall or recurrence-free survival, the quality of the survival analyses in several studies is questionable.

Specifically, three articles performed survival analysis using incorrect methods, while one study did not conduct a survival analysis at all. Moreover, three studies reported risk ratios instead of HR, which is inconsistent with the appropriate methodology for survival analysis.

Furthermore, there is currently no gold standard for the detection of VM in NSCLC. All studies used double staining with CD31/CD34+PAS, but there are several mimics and pitfalls that complicate the differential diagnosis of VM. In some articles, the structures described as VM by the authors raise doubts about their true nature.

Based on the results of our systematic review, we conclude that VM is significantly associated with poor prognosis in patients with non-small cell lung cancer. Multivariate analyses across multiple studies demonstrated that VM serves as an independent negative prognostic factor, increasing mortality risk by 1.5-2.7 fold and disease progression risk by 2.1-2.4 fold. However, these findings must be interpreted with caution due to substantial methodological limitations identified in the available research.

The true prevalence of VM in NSCLC remains unclear, and further research is needed to establish a standardized approach for VM detection and to validate its prognostic significance in larger, prospective cohorts with diverse ethnic backgrounds. Future studies should adhere to rigorous methodological standards, including appropriate survival analysis techniques and transparent reporting of results, to provide more reliable and conclusive evidence regarding the role of VM in NSCLC prognosis.

## Data Availability

The original contributions presented in the study are included in the article/supplementary material, further inquiries can be directed to the corresponding authors.
